# Scoping Review: Suicide Specific Intervention Programmes for People Experiencing Homelessness

**DOI:** 10.3390/ijerph18136729

**Published:** 2021-06-22

**Authors:** Rachael McDonnell Murray, Eilis Conroy, Michelle Connolly, Diarmuid Stokes, Kate Frazer, Thilo Kroll

**Affiliations:** 1 The Department of Clinical Psychology, School of Health in Social Science, The University of Edinburgh, Edinburgh EH8 9AG, UK; 2 The Academic Unit of Neurology, Trinity College Dublin, Dublin D02 PN40, Ireland; econroy16@gmail.com; 3 Dublin Simon Community, Dublin D07 PD37, Ireland; michelleconnolly@dubsimon.ie; 4 University College Dublin Library, University College Dublin, Dublin D04 V1W8, Ireland; Diarmuid.stokes@ucd.ie; 5 School of Nursing, Midwifery and Health Systems, University College Dublin, Dublin D04 V1W8, Ireland; kathleen.frazer@ucd.ie (K.F.); thilo.kroll@ucd.ie (T.K.)

**Keywords:** suicide prevention, homelessness, scoping review, inequalities in health, poverty, mental health

## Abstract

Background: The homeless population are among the most vulnerable groups to experience suicide ideation and behavior. Several studies have shown that people who are homeless experience more significant suicidal ideation and behavior than the general population. However, there is limited information about what suicide interventions exist, to what extent they are grounded in robust research, and which intervention components effectively reduce suicidal ideation and behavior in the homeless community. This research aimed to characterise the current evidence base in the area of suicide prevention for homeless individuals. Methods: A scoping review guided by Arksey and O’Malley’s five-stage framework was conducted and a narrative synthesis was performed. Pubmed, EMBASE, PsychInfo, Cumulative Index to Nursing and Allied Health Literature (CINAHL), Open Grey, and Bielefeld Academic Search Engine were searched up to 8 May 2020. Results: A total of 3209 records were identified through database and grey literature searching. Three studies are included in this review. Key outcomes identify suicide intervention prevention programmes; similarities and differences across interventions, and examples of staff training. A quality review of the studies was completed. Conclusion: A stark gap in the evidence of suicide specific prevention interventions targeted at homeless populations.

## 1. Introduction

Suicide and suicidal ideation are recognized as a public health issue by the World Health Organisation (WHO) [1] and are a vital target of the Sustainable Development Goals [2]. Globally, 800,000 people die annually by suicide, and it is estimated that for each suicide, there are more than 20 attempts [3]. Suicide rates are particularly high in Europe and South East Asia—15.4 and 13.2/100.000, respectively for both genders and are lowest in the Eastern Mediterranean region (3.9/100.000). However, suicide may not always be recorded in some parts of the world due to stigmatization [4]. Rates of suicide are higher in males, and in Europe specifically, they are up to four times higher when compared to women [3]. It is the third leading cause of death worldwide in those aged 15 to 29 years; however, age differences exist between high, low and middle-income countries. The WHO [5] confirms that younger aged adults and older women in low and middle-income countries (LMICs) have higher rates of suicide when compared to higher-income countries; middle-aged men in high-income countries have increased rates when compared to counterparts in LMICs. However, data reporting is limited, and the WHO [4] acknowledges that only 80 of their 183 member states have quality reporting systems in place.

Risk factors associated with suicide, suicidal ideation, and behaviors are fourfold: individual, relationship, community and societal [5]. They are heterogeneous and highly variable. Mental illness, social isolation, financial problems and substance use disorders are associated with increased individual risk. Relationship factors increasing risk include adverse childhood experiences, bullying, family history of suicide and relationship problems, including violence. Finally, community and societal risks include barriers to care, cultural beliefs, the stigma associated with seeking help, media portrayal and access to means and resources, e.g., firearms, pesticides [5,6]. While geographical differences exist, Turecki and Brent [6] suggest cultural factors hold a more significant impact, as the *suicide rates of immigrants are more closely correlated with their country of origin than with their adoptive country* (p. 2).

Homelessness is a significant public policy and health service concern in many countries, impacting up to 1% of the global population [7]. Fazel et al. [8] report 400,000 individuals are homeless on any one night in the European Union, and more than 600,000 are homeless in the USA. In the UK, data estimates the homeless population range from 0.26% to 1.5% of the total population in each region; the highest rates are reported in Scotland. In Ireland, 0.13% of the population, 6000 adults, are identified as homeless; a further 2326 children are homeless [7,9].

There is no agreed definition of ‘homelessness’ and according to the European Typology on Homelessness and Housing Exclusion (ETHOS), it can *“include rooflessness (without a shelter of any kind, sleeping rough), houselessness (with a place to sleep but temporary in institutions or shelter), living in insecure housing (threatened with severe exclusion due to insecure tenancies, eviction, domestic violence) and living in inadequate housing (in caravans on illegal campsites, in unfit housing, in extreme overcrowding)”* [10]. Causes of homelessness are complex and can be linked to profound mental health challenges, either as a contributing factor to becoming homeless or as a consequence of living without a home [8].

For those who are homeless, consistent research reports risk factors for suicidal ideation and behaviors such as excess unemployment [11], early adverse childhood experiences [12], substance misuse [13], and lack of perceived social and emotional support [14]. Moreover, risk of attempting and completing suicide increases due to stressful life events, chronic physical illnesses, and untreated mental illnesses [15,16,17].

Suicidal ideation and behavior among people who are homeless are substantially elevated compared to the general population. Research by Tsai and Cao [18] reveals that attempted suicides could be up to 5.3 times higher among individuals who experience homelessness than the general population. Sinyor et al. [19] also report disproportionately high rates of suicide among their sample of participants who were homeless, suicide deaths among the homeless population were 10-fold, compared with the general population.

Barrett et al.’s [20] study of an Irish registry reported incidence rates of self-harm presenting to hospital emergency departments were up to 30 times higher among the homeless population than those living at a fixed residence during the period 2010 to 2014.

Homeless individuals often present with a myriad of complex needs that increase their risk of suicide, such as a long-standing history of addiction issues, traumas, loss, financial burden, and mental and physical illness [21]. Many individuals among the homeless community have limited social support and face several barriers when accessing mental health services, such as stringent entry criteria and transport issues [22]. Despite this, there is limited focus on the intersectionality of homelessness and the effects of race, class and gender [23], including mental illness and the increased risk of suicide and suicide ideation. In a review of the current evidence-based suicide prevention programmes for culturally diverse groups, including homeless individuals, Poon [24] concluded that the majority of existing suicide prevention interventions are not specifically tailored to a culturally diverse group’s need.

There is a growing body of evidence emerging to understand suicidal ideation and behavior in greater depth, and significant efforts made in developing effective suicide specific treatment, and preventative interventions for the general population. Dialectical Behavioural Therapy [25], Cognitive Behavioural Therapy (CBT) [26], Brief CBT for suicidal risk [27], the Collaborative Assessment and Management of Suicidality [28,29], and the Attempted Suicide Short Intervention Programme [30] are the primary approaches for which there is evidence that they can be efficacious in reducing suicidal ideation and preventing suicide. 

While suicide-specific interventions for people who are homeless are desirable, there has been no evidence review that identifies the scope, nature and efficacy of such interventions [31]. Therefore, this scoping review aims to characterise the state-of-the-science in this area, uncover best prevention, counselling, and treatment practices, and identify any further research necessary.

This review’s specific objectives are to (1) systematically review published research in scope, nature, and quality, and (2) identify gaps that may currently permeate the literature. Additionally, components of interventions delivered in clinical settings, which are most effective in reducing suicidal ideation and behavior among the homeless population, will be identified and presented.

## 2. Materials and Methods

We developed a protocol for this scoping review using the five-stage methodological framework guided by Arksey and O’Malley [32]: (1) Identifying the research question; (2) identifying relevant studies; (3) study selection; (4) charting the data; and (5) collating, summarizing, and reporting the results. The protocol was developed a priori and is included in Supplementary Materials File S1. We report our findings according to the Preferred Reporting Items for Systematic Reviews and Meta-Analyses Scoping Review (PRISMA-ScR) checklist [33] see Table 1 below.

### 2.1. Review Question

The scoping review aimed to answer the following question: What is the extent, scope, effectiveness and context of suicide specific intervention programmes for individuals who are homeless? 

### 2.2. Search Strategy

A systematic search strategy was developed in consultation with an expert librarian (DS). We systematically searched four electronic databases from inception up to May 8 2020: Pubmed, EMBASE, PsychInfo and Cumulative Index to Nursing and Allied Health Literature (CINAHL). Open Grey and Bielefeld Academic Search Engine were used to search for grey literature.

Three concepts were used in the search “suicide specific intervention”, “homelessness”, and “suicide”. The systematic and comprehensive search strategy consisted of key search terms derived from existing search strings and bespoke for each electronic database. The search terms were as follows: (“suicide”* OR “self-harm” OR “deliberate self-harm” OR “self-injurious behavior*” OR “suicidal ideation” OR “mentally ill” OR attempted suicide”) AND (“homeless*” OR “no fixed abode” OR “rough sleeper” OR “homeless person” OR “person, homeless” OR “street people” OR “people, street”) AND (“suicide intervention*” OR “psychotherapy” OR “cognitive psychotherapy” OR “treatment” OR “crises intervention service*” OR “evidence based practice” OR “mental health service” OR “management” OR “measurement” OR “assessment” OR “cognitive therapy” OR “health care utilization” OR “emergency services” OR “collaborative care” OR “prevention” OR “suicide prevention”) (note: * indicates a wildcard). The results were combined using Boolean operations and adapted for each database (see Supplementary Materials File S1). Grey literature sources were hand-searched along with conference proceedings, LENUS, and thesis databases. Major journals in this field were also hand searched (European journal of Homelessness, PROQUEST). We also scanned references of the included articles for any relevant studies.

### 2.3. Eligibility Criteria

Assessment criteria were developed to guide the review and inclusion of papers are presented in Table 2. Articles were included if they were peer-reviewed and no time restrictions were imposed on any paper, but language was restricted to papers published in English.

### 2.4. Study Selection

Search results were imported into the Covidence software management system [34] for screening by multiple reviewers. Level 1 screening focused on inclusion criteria based on titles and abstracts, while level 2 screening involved reviewing full-text articles.

Three reviewers [RMcDM, EC, KF] independently screened all titles and abstracts. Reviewers met throughout the screening process to discuss queries and reduce uncertainties. The full-text screening was completed independently by three reviewers [RMcDM, EC, KF], with disagreements resolved with discussion with reviewers and TK.

A standardized data extraction template was developed in Excel following a review of previous templates used by members of the team. The extraction form was tested by two independent reviewers [EC, MC] and checked by a third reviewer [RMcDM] for completeness and reliability. Following discussions with reviewers [RMcDM and KF] extraction proceeded independently by two authors [EC, MC] for included articles. Data extracted included: Author(s), year of publication, study location, study design, intervention description, study sample or population characteristics, aims of the study, methodology, description of the reported intervention, outcome data including a suicide attempt, suicidal ideation, mortality, suicide-related behavior, and/or self-harm at the point of post-intervention and duration of follow up period.

### 2.5. Methodological Quality Appraisal

The methodological quality of included articles was assessed using the Mixed Methods Assessment Tool (MMAT) [35]. The MMAT is used widely and considered a valid indicator of methodological quality for non-randomized and descriptive studies. Two review authors [EC & MC] independently assessed the quality of included studies with disagreements resolved by a third author [KF], and were discussed with [RMcDM] (Supplementary Materials File S2).

### 2.6. Data Summary and Synthesis

Data from included studies were collated, and Table 3 presents characteristics and methodologies reported. Table 4 outlines evidence of informed interventions. A narrative analysis describes and synthesises the scope, nature and effectiveness of informed interventions.

## 3. Results

### 3.1. Literature Search

**Figure 1:**
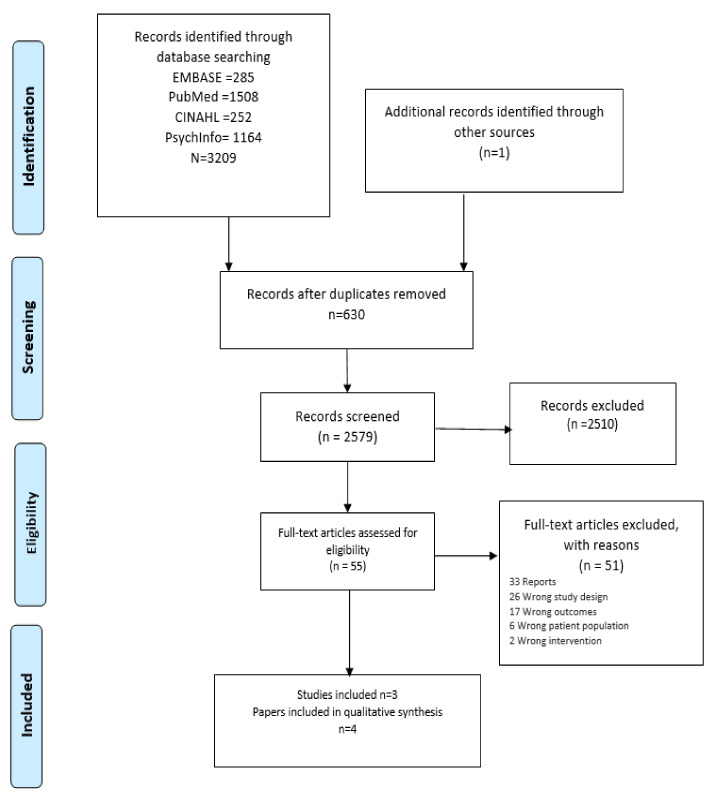
PRISMA 2009 Flow Diagram.

A total of 3209 records were identified through database searching; one additional article was included from the grey literature search (Figure 1). After deduplication, 2579 records were screened by title and abstract. After screening, 55 potentially relevant full-text articles were reviewed. Fifty-one articles were excluded, as they did not meet the inclusion and exclusion criteria (Table 1). Articles were excluded on the basis that (1) they did not include an intervention specifically for the population under review’ people experiencing homelessness, (2) they did not report empirical research, and (3) reported outcomes did not meet the inclusion criteria for this review.

### 3.2. Study Characteristics

Three studies are reported in representing four papers (Table 2). Slesnick et al. [16] and Wu et al. [36] are geographically located in the US and report outcomes following a randomized controlled trial. Lynn et al. [37] study was also located in the US, and Adams et al. [38] was based in Ireland. The study populations consist of adult participants and staff members [38], adolescents and their parents [37] and young adults aged 18 to 24 years [16,36]. All studies report randomized controlled trial methods, except for Adams et al. [38] reporting a mixed-methods study (Table 2).

### 3.3. Analysis

Characteristics and data from included studies were grouped, and evidence is presented in a narrative summary reporting best practices of prevention, including counselling and treatments.

### 3.4. Characteristics of Participants

The identified studies addressed suicidal ideation and behaviors in adolescents as well as in adults, encompassing youths aged 11 to 14-year-olds [36], young adults aged 18 to 24 years [16,37], and adults above the age of 18 [38]. Previous suicide attempts were reported in two of the studies. Adams et al. [38] reports 64% of participants had a prior suicide attempt, Wu et al. (36) and Slesnick et al. [16] reports 80% of participants had a previous suicide attempt) (Table 2).

### 3.5. Characteristics of Intervention Programmes

The type and duration of interventions varied (Table 2). Adams et al. [38] detail a Collaborative Assessment and Management of Suicidality programme consisting of 3 to 13 sessions. Lynn et al. [37] included (1) a family-focused programme intervention completed over eight sessions of 16 h duration, and (2) a health education programme comprising three sessions of two-hour duration. The study intervention of Slesnick et al. [16] and Wu et al. [36] was a cognitive therapy for suicide prevention programme (10 sessions × 50 min) with a further option of attending nine subsequent sessions. Follow up of participants in this study was at three monthly intervals up to nine months post-intervention.

### 3.6. Suicide Intervention Prevention Strateiges

The intervention programmes are described (Table 2 and Table 3). Adams et al. [38] describe an established Collaborative Assessment and Management of Suicidality (CAMS) programme providing a therapeutic framework for clients experiencing suicidal ideation to work with practitioners collaboratively to assess the risk of suicide for planning and managing behaviors using a Suicide Status Form. It is a theoretically based flexible programme facilitating a tailored approach to meet individual needs. Participants engaged with CAMS over several months with one of four trained facilitators in several city-center locations. Completing the programme was deemed successful if no suicidal thoughts, feelings and behaviors were reported at three consecutive sessions.

Wu et al. [36] and Slesnick et al. [16] described a Cognitive Therapy for Suicide Prevention (CTSP) and compared the intervention to ‘treatment as usual’. The CTSP is additional to the usual care and was provided in three-phased blocks over 10 sessions. The intervention programme is theoretically underpinned in cognition and behavioral response. During the initial phase of treatment (sessions 1–3), clients were educated on the cognitive model, and a cognitive case-conceptualization developed based on the client’s risk-factors and experiences. A crisis plan was developed during the first session. The middle phase of treatment (sessions 4–7) focused on cognitive restructuring and behavior change to address suicide-specific risk factors and includes therapies such as distraction, relaxation, and intense physical sensations. The objective of the later sessions (8–10) was prevention through practicing skills using guided imagery. An open-door policy was used to support participants with no appointments necessary to access a practitioner. An incentive of a $5 food gift card was provided for each attendance.

Lynn et al. [37] described a HOPE programme consisting of two elements; a family programme, an eight-session of weekly composed of one-hour meetings that included separate and conjoint sessions for parents and young people attending. This programme enabled free discussion among peers and then as a family unit. The programme targeted intensive family support, including communication, parental monitoring, supervision skills, and stressful situations.

The HOPE health educational programme provided information sessions to prevent HIV/AIDS and sexually transmitted infections, use of illicit substances and normative adolescent changes. The programme was delivered over three sessions of two-hour duration in separate groups for caregivers and young adults. A social worker facilitated both sessions, supported by community members experienced in HIV prevention services, and the intervention programme was delivered in specific family shelters. The shelters were randomized to either programme.

### 3.7. Similarities and Differences across Suicide Prevention Approaches

Studies report findings from three different suicide specific interventions. One consistency reported across all interventions employed was a focus on relationships and the clients’ social network. This was emphasized in the studies conducted in adolescents and young adults [16,36,37]; limited data are reported by Adams et al. [38] on family relationships or social supports. It was one of the main reasons for living indicated by clients and one of the primary reasons for wanting to die—no information reported on the impact of the intervention on relationships in the clients’ lives.

In contrast, Lynn et al. [37] reported that the HOPE Family Programme was 13 times more likely to report a decrease of suicidal ideation compared with the education-only approach. The authors speculate that the intense focus on family processes, communication, and coping skills may have contributed to the approach’s effectiveness. Similarly, Wu et al. [36] and Slesnick et al. [16] note that the CTSP was effective in reducing cognitive distortions of social alienation and associated suicidal thoughts; family network satisfaction was highlighted as a core factor in conceptualizing homeless youth’s suicidal ideation and was reported to have led to enhanced treatment effects [36] (Table 3).

### 3.8. Staff Training

Two studies report staff training before implementing the intervention [16,36,38], with only one study reporting on fidelity to the intervention throughout treatment [16,36]. The CTSP training consisted of readings and a three-day on-site training in the intervention, which included role-play exercises. Dr. Wenzel (the developer of the programme) provided ongoing weekly telephone/skype supervision. Similarly, the CAMS training consisted of engaging in a 3-h online learning module, a one-day live role-play workshop, and follow up case consultation phone calls [38]. No fidelity ratings were reported for this study (Table 2).

### 3.9. Challenges in Accessing and Using Suicide-Specific Interventions

All three studies highlight a need to remove barriers for youth and adults to have access to mental health services. Such barriers include the cost of accessing services, lack of insurance by many homeless clients, difficulty accessing services due to their location, and the lack of transportation. Adams et al. [38] identified that services were required outside the restricted hours of 9–5 pm, usual for homeless clients. Lynn et al. [37] recommended that programmes target the complexities of care for homeless clients mental health, substance abuse prevention, HIV/STD prevention (Table 3).

### 3.10. Quality Review

While scoping reviews strictly do not require a systematic quality appraisal, we applied the Mixed Methods Assessment Tool (34) [See Supplementary Materials File S2]. We did not exclude any studies based on their quality assessment. While two studies report randomizations, this referred to randomization of one of two programmes for participants attending a HIV outreach programme and living in 28 of 204 family shelters [36]. Participants recruited by Slesnick et al. [16] and Wu et al. [36] were approached while attending a local drop-in center and assessed for eligibility. They were randomized to either the intervention programme or usual care.

Convenience sampling was evident in all studies with small sample sizes reported. The loss to follow up and completion was highest in Adams et al. [38], reporting four of seventeen participants completing the programme. While Lynn et al. [37] randomized 28 shelters to one of two programmes, selecting the 28 from the 204 urban shelters is not described.

Training of staff involved in the delivery of the intervention programmes was reported by Adams et al. [38] and comprised completing a 3-h online learning module, a one-day live role-play workshop and follow up case consultation phone calls. Slesnick et al. [16] and Wu et al. [36] detailed a training programme including recommended readings and a three-day on-site training in the intervention, including role-play exercises by one of the original developers. There was ongoing weekly support for therapists. Three studies identified that therapists were qualified professionals and/or educated up to the Masters level. Lynn et al. [37] reported that facilitators were highly experienced community members with more than five years of experience in HIV services. While all studies present evidence of impact of intervention programmes on reducing risks of suicidal ideation, the family HOPE intervention programme did not assess risk factors [35].

Baseline and outcome variables were measured using standardized but self-reporting instruments. Slesnick et al. [16] and Wu et al. [36] completed follow up data collections at 3-, 6-, and 9-months post-intervention retaining 86.6% of participants in both intervention and usual care groups (N = 75 at baseline). The inclusion of a financial incentive was provided for each session attended, which may have influenced participation.

Self-reported data was collected and is associated with recall bias. In addition, Wu et al. [36] acknowledged employing a total scoring of assessment and identified this may not be representative of individual family members.

## 4. Discussion

### 4.1. Main Findings

This scoping review presents, for the first time, a limited evidence base for suicide specific prevention programmes developed and tailored for homeless population groups following a systematic search of databases. The current evidence provides examples of intervention programmes primarily focusing on youths and young adults, in community services, and illustrates the impact of intervention programmes on reducing suicidal ideation and risk of suicide. Three intervention programmes described the benefits to participants, families, and employees. The dearth of intervention programmes for this marginalized group is stark.

In the past decade, there have indeed been important developments in the understanding and prevention of suicide. There has been an exponential increase in empirical support for the treatment of suicidal risk [39], and greater efforts made to raise awareness and provide basic training to engage the public in recognizing and speaking openly about suicide [40,41]. Despite public health efforts, the extent to which these developments have reduced rates of suicidal ideation, attempts or completed suicides among the homeless population is not clear.

It has been recognized across the evidence base that risk factors of suicidal ideation and behavior among people who are homeless are substantially elevated compared to the general population; with research reporting suicide attempts can be up to 5.3 times higher among individuals who experience homelessness compared with the general population [19]. Despite this, the current review highlights the significant gap in implementing and evaluating such interventions among the homeless population.

Most efforts to reduce suicidal ideation and behavior among people who are homeless rely on indirect approaches to prevent suicide. These include strategies to enhance mental health through the reduction of anxiety and depression, enhancing perceived self-efficacy and empowerment [42], or through interventions such as Housing First (HF). Of course, an ongoing effort to address underlying housing insecurity and to tackle primary social determinants leading to poverty should be prioritized. HF for example, is a strong example of an intervention which aims to tackle the issues which leads to and perpetuates the many difficulties that people who are homeless face. However, the evidence is mixed on whether HF improves the complex mental health needs of those who are homeless compared with treatment as usual. Most studies have found that although HF is an essential intervention to provide housing stability and has been shown to increase quality of life and decrease hospital stays, it does not show greater improvements in mental health scores or reduce suicidal ideation or attempts any more than treatment as usual [42,43].

As noted by Aquin et al. [44] traditional community resources are not designed, nor equipped to address the unique risk factors of homelessness, therefore without relevant training and tailored interventions, those who are having suicidal thoughts or behavior, are directed to emergency services. Therefore, treatment as usual for many homeless individuals who are experiencing suicidal ideation or behavior, is often attending emergency services. Although there is a necessity for hospitalization in high suicidal risk situations, to date there is no conclusive evidence to suggest that hospitalization is an effective form of treatment for suicidal patients [39,45].

Taken together, although such indirect strategies have shown efficacy in tackling mild mental health difficulties and risk factors related to suicide individuals who are suicidal and only receive indirect support or usual care are more likely to die by suicide when compared with those receiving direct, i.e., suicide-specific interventions [46]. In contrast, research conducted with clients treated with suicide-specific interventions reveals significantly greater post-treatment improvements and medium improvements at longer-term follow-up when compared with clients treated with indirect strategies [47]. The growth of clinical trials on suicide-specific interventions has been highlighted as a salient development in suicide research and practice, and it has been unanimously acknowledged by experts in the field of research and prevention, that suicide must be the focus of treatment rather than viewing it as a symptom of some other mental disorder [48].

The complex health issues associated with marginalized groups including people experiencing homelessness are known, with increased risk of ill health and underuse of healthcare services. Robards et al. [49] identifies access as a social determinant of health, and people experience barriers due to socioeconomic, social or cultural reasons (p.2). 

The three studies in this review did not capture the cultural identity of the participants; only the location in which the studies is known. Tailoring interventions to the needs of diversity, equality and inclusion amplifies the challenges faced by services [24]. However, research by Poon [24] revealed that although the homeless population are a diverse group, homelessness in itself may be considered a shared cultural identity, regardless of their other intersecting identities such as gender identity, sexual orientation, ethnicity [50]. Future research studies must capture information on cultural identitites and gain more of an understanding of the specific needs of minority groups within the homeless population.

Many individuals who are homeless have limited social support and face several barriers when accessing mental health services, such as inflexible entry criteria and transport issues [23]. Culatto et al.’s [51] 16-year retrospective review of suicide rates in homeless patients in England and Wales, confirmed increasing risks associated with chronic alcohol, drug and substance misuse than for patients in stable accommodation. Homeless patients were reported as more likely to die as in-patients (21% V 10%) or within three months of discharge (32% V 19%) compared to housed counterparts. It is evident from this data, that there is a need to focus on the intersectionality of homelessness, including mental illness, and risk of suicide and suicidal ideation [24] when considering intervention programs.

Furthermore, Robards et al. [49] notes intersectionality is an approach to understand multiple health inequalities and is an important framework for understanding the social determinants of health. This view is articulated recently by Marteau et al. [52] seeking a focus on social determinants of health to address health inequities, more visible during the COVID-19 pandemic. The pandemic has noticeably widened the gap between the rich and poor and exacerbated pre-existing social, economic, and political inequalities, including inequalities of wealth, health, well-being, social protection and access to basic needs such as food, housing, healthcare and, schooling [53].

Attempting to address inequalities in suicide prevention by taking a ‘fire fighter’ approach, is not effective, the interaction of variables which produce health inequalities to begin with must be addressed first [54]. Hochhauser et al. [55], notes that suicide is still largely seen as an entirely personal matter, despite the fact that suicide, can and often is, a response to external conditions that are outside of an individual’s immediate control. Public health strategies, allowing for an in-depth and non-judgmental understanding of the reasons for suicide prevalence in specific communities and minority groups, based on a social justice framework, are needed. Specifically, health care providers, educators, politicians and the general public must be made aware of how socioeconomic inequality cultivates poverty, homelessness, racism, health inequalities, discrimination, historical trauma, and the extent to which this can contribute to suicidal ideation and attempted suicide, in marginalized and minority groups [55,56,57]. Socioeconomic risk factors for suicide are structurally reinforced, and it is imperative, going forward, we address systematic social disadvantage and injustice from occurring.

The current evidence presents the impact for families when supported especially the use of cognitive behavioral therapies [16,36]. However, Lynn et al. [37] and Slesnick et al. [16] confirm the need to know more about this community and especially those not accessing supported services. Adams et al. [38] acknowledges the benefits identified by staff to their learning, but limited insight beyond. What is evident is the gaps in services and identifying the critical need for support for such marginalized groups. An examination of the salient gaps in the research and within practice, highlights several opportunities.

### 4.2. Opportunities

Poor communication within the family is a crucial risk factor for suicide among adolescents in the general population [58]. Providing strategies to strengthen family relationships are recommended elsewhere [59] to reduce suicidal ideation and behavior. One of the main mechanisms of change speculated for children and adolescents was family network satisfaction, communication, and coping skills [16,36]. Therefore, effective prevention interventions should continue to focus on connecting service users, and the development of parenting skills, and increasing social support for service users [25].

Working with individuals who are homeless and are at risk of suicide is an anxiety provoking experience, and in order to support frontline workers, or mental health professionals in implementing prevention strategies or interventions, training is required [35]. In this review, staff training was reported in-depth by Slesnick et al. [16].

Adams et al. [38] provided data on learning pre and post the introduction of a CAMS program. Follow up research to this study, which explored counsellors’ experiences of implementing CAMS within the service, identified the lasting impact that training had [60]. McDonnell Murray et al. [60] noted that many of the counsellors reported they developed more direct approaches of speaking about suicide. that The CAMS and the SSTT provided counsellors with confidence in assessing suicidal risk, and for structuring sessions. 

Equally, Poon [24] considers training a priority for staff using suicide specific preventative strategies. Ensuring practitioners receive adequate training, sufficient supervision and explore their experiences implementing or adapting such interventions, are necessary steps to ensure the quality of care provided to service users and for the well-being of staff [61].

Furthermore, drawing on evidence in this review and identifying specific challenges in accessing and using suicide specific interventions among the homeless population, provides a clear argument to involve service users and experts by experience in the development and evaluation of such interventions [62]. Experts by experience, service users, and stakeholders, can help explicitly enhance the relevance, acceptability, effectiveness and sustainability of treatments [63,64].

### 4.3. Implications for Practice and Future Research

This scoping review identifies gaps in suicide specific interventions designed for the homeless populations and guides the focus of future research studies. A continued effort is needed to reduce poverty and provide housing for those at risk of homelessness; however, addressing the current mental health needs of the homeless community, which are highly prevalent, should prioritised. Despite this being recognized as a priority previously, with international targets of the WHO’s [65] Comprehensive Mental Health Action Plan to reduce suicide-related mortality by 10% between 2012 and 2020, it was not achieved. In Ireland, like in other countries, people who are homeless are included as a priority group due to their greater vulnerability in national planning efforts to mitigate the risks of attempting suicide [66]. However, evidence from this scoping review indicates the limited targeting of homeless individuals to participate in suicide prevention programs.

All three studies highlight barriers for youth and adults’ access to mental health services, including the financial cost of accessing services, lack of insurance by many homeless clients, difficulties of access due to location, and a lack of transportation. Services are required beyond the hours of a 9 to 5 service [37]. Brownson et al. [67] identified, race-based discrimination through one system, (in this case housing/insurance/transportation) is reinforced in interlocking systems such (access to mental health care), and how these systems can in practice undercut the effectiveness of interventions developed in rigorous, and controlled efficacy studies. As highlighted above, a systems-based approach to tackle institutional discrimination is required to achieve health equity [68].

Services must also be able to cater for the complex needs of homeless clients [35]. Additionally, dual diagnosis presents an added layer of complexities for service providers, and often, staff do not feel equipped to meet these needs without sufficient input from psychiatry [59,69]. With such complexities in mind, accessibility and availability must be at the forefront of developing and providing mental health services to homeless populations.

Planning and co-development of future research should commence by asking why limited evidence exists. A health systems lens is necessary to identify root causes, relationships, and interdependent parts, including pressures, policies and power dynamics, to understand the complexity and context of service provision [70]. Consideration of multiple actors and diverse stakeholders involved in care services, including homeless services, mental health services, addiction services and primary care services, developing common ground is vital with the person and family at the centre of all planning [70].

Including user-developed participatory studies are required [70]. Poon [24] included clients and staff as participants in their qualitative study and critical factors identified a need for staff training, client monitoring among staff; and a lack of social support, and feelings of hopelessness from clients. Evidence within this current review presents only objective data obtained from the completion of multiple survey instruments. The voice of clients and families is absent. There is a question to consider who benefits most from asking marginalized groups to engage and complete multiple survey instruments. Who is also missing from studies, including those who have literacy and language barriers. Perhaps these studies exist and are not published or available. This emphasizes a need for a re-evaluation of how research in this context is conducted. Clear guidance exists which outlines the best possible strategies of evaluating complex interventions, whilst still retaining research integrity and quality. Such strategies may include the development of research that requires a less linear model of evaluation, research which places more emphasis on the integration of process and outcome evaluation, and which is specifically tailored to local services [71,72]. Future funding must include design thinking methods, ensuring a human centered approach [73,74].

Finally, there needs to be a greater effort to make health equity the focus and central aim of implementation science. Brownson et al. [67], outlines ten steps of action required to ensure that resources are invested in health-related research to eliminate disparities to achieve health equity.

### 4.4. Strengths and Weaknesses

A strength of this review is that it was conducted using a predefined protocol adhering to Arksey and O’Malley’s framework [32]. Studies were included following review against an eligibility criteria and independent assessment by two reviewers for screening and data extraction. The quality of evidence was assessed independently by two reviewers, which is not a requirement of a scoping study. Inclusion of grey literature sources is a strength; we contacted authors to seek further information on papers and dissertations. Registering the review prior to conducting the search would have been helpful. Few limitations were imposed on the review; we did not restrict on study design or age of included populations. Our review is limited in that evidence was included if published in English only, it is likely that other relevant studies from non-English speaking countries could be excluded on this basis.

## 5. Conclusions

This review found a limited number of studies evaluating suicide-specific interventions for homeless populations. Three themes identified across the studies include: similarities and differences across interventions, the importance of formal training for staff, and the quality of the research which has been conducted to date. For future research and implementation of suicide specific intervention programs, experts by experience, staff and service users should all be consulted with and included to collaboratively co-design interventions and evaluations. Safe and sustainable housing is fundamental to the physical, mental and social wellbeing. Significant political will and targeted resources are required to end homelessness, and while it is a complex challenge, it is possible. Sustained investment in adequate housing is of critical importance. However, many countries are at a considerable distance from being in a position to end homelessness, it is imperative that any strategy to end homelessness must also address the adverse impact of not having a home in the interim. Evidence from this review warrants the immediate provision of effective mental health services, implementing suicide interventions that are accessible and available to people experiencing homelessness, in addition to approaches such as HF to provide stable housing. A central focus for such interventions should be placed on strategies to strengthen family relationships, communication skills, and to develop coping skills. Providing formal suicide specific training which meets the needs of the service is necessary for all professionals working with individuals who are homeless and who are presenting with suicidal ideation and behavior. Finally, health equity should be a key factor to hold at the forefront of implementation science, in the design and development of policies, services and in the allocation of resources. There is a critical urgency to address the widening gap of health inequality on a national, and international level.

## Figures and Tables

**Table 1:** Preferred Reporting Items for Systematic reviews and Meta-Analyses extension for Scoping Reviews (PRISMA-ScR) Checklist.

Section	Item	Prisma-Scr Checklist Item	Reported on Page #
**Title**			
Title	1	Identify the report as a scoping review.	1
**Abstract**			
Structured summary	2	Provide a structured summary that includes (as applicable): background, objectives, eligibility criteria, sources of evidence, charting methods, results, and conclusions that relate to the review questions and objectives.	3–5
**Introduction**			
Rationale	3	Describe the rationale for the review in the context of what is already known. Explain why the review questions/objectives lend themselves to a scoping review approach.	1–3
Objectives	4	Provide an explicit statement of the questions and objectives being addressed with reference to their key elements (e.g., population or participants, concepts, and context) or other relevant key elements used to conceptualize the review questions and/or objectives.	3
**Methods**			
Protocol and registration	5	Indicate whether a review protocol exists; state if and where it can be accessed (e.g., a Web address); and if available, provide registration information, including the registration number.	N/A
Eligibility criteria	6	Specify characteristics of the sources of evidence used as eligibility criteria (e.g., years considered, language, and publication status), and provide a rationale.	4
Information sources	7	Describe all information sources in the search (e.g., databases with dates of coverage and contact with authors to identify additional sources), as well as the date the most recent search was executed.	4
Search	8	Present the full electronic search strategy for at least 1 database, including any limits used, such that it could be repeated.	4
Selection of sources of evidence †	9	State the process for selecting sources of evidence (i.e., screening and eligibility) included in the scoping review.	4
Data charting process ‡	10	Describe the methods of charting data from the included sources of evidence (e.g., calibrated forms or forms that have been tested by the team before their use, and whether data charting was done independently or in duplicate) and any processes for obtaining and confirming data from investigators.	5
Data items	11	List and define all variables for which data were sought and any assumptions and simplifications made.	5
Critical appraisal of individual sources of evidence §	12	If done, provide a rationale for conducting a critical appraisal of included sources of evidence; describe the methods used and how this information was used in any data synthesis (if appropriate).	5
Synthesis of results	13	Describe the methods of handling and summarizing the data that were charted.	5
**Results**			
Selection of sources of evidence	14	Give numbers of sources of evidence screened, assessed for eligibility, and included in the review, with reasons for exclusions at each stage, ideally using a flow diagram.	5–6
Characteristics of sources of evidence	15	For each source of evidence, present characteristics for which data were charted and provide the citations.	Table 2 and Table 3
Critical appraisal within sources of evidence	16	If done, present data on critical appraisal of included sources of evidence (see item 12).	N/A
Results of individual sources of evidence	17	For each included source of evidence, present the relevant data that were charted that relate to the review questions and objectives.	6–11
Synthesis of results	18	Summarize and/or present the charting results as they relate to the review questions and objectives.	Table 2 and Table 3
**Discussion**			
Summary of evidence	19	Summarize the main results (including an overview of concepts, themes, and types of evidence available), link to the review questions and objectives, and consider the relevance to key groups.	17
Limitations	20	Discuss the limitations of the scoping review process.	21
Conclusions	21	Provide a general interpretation of the results with respect to the review questions and objectives, as well as potential implications and/or next steps.	21
**Funding**			
Funding	22	Describe sources of funding for the included sources of evidence, as well as sources of funding for the scoping review. Describe the role of the funders of the scoping review.	22

† ‡ Details in full Section 2.2, Section 2.3 and Section 2.4. § Mixed Methods Assessment Tool used. # Page number.

**Table 2:** Eligibility criteria for selection of publications.

Inclusion Criteria	Exclusion Criteria
1. Eligible study populations were composed of children, adolescents, adults and older adults who were homeless.	1. Studies were excluded if they were not carried out on a homeless population.
2. All studies were required to be in the English language.	2. Studies were excluded if published in another language.
3. Eligible studies were required to be empirical research that evaluated suicide specific interventions. Grey literature, case studies, unpublished theses, peer-reviewed journal articles, articles that have not been peer-reviewed were all included.	3. Opinion/theoretical papers. Interventions that were not explicitly designed for reducing suicidal ideation and behavior.
4. Eligible studies included those which measured suicidal ideation and behavior following the intervention.	4. Studies were excluded if they did not include the evaluation of an intervention.

**Table 3:** Study Characteristics.

Author Year of Publication	Study Location	Study Design	Population Characteristics	Intervention Description	Aims of the Study	Methodology
Adams et al. 2018	Dublin, Ireland A charitable organization Dublin Simon Community. The study was conducted in two residential short-term accommodations and one medium supported housing unit.	Mixed methods pilot study	1. Clients/Service Users. The sample consisted of 17 adult participants who were homeless and who were referred to the Sure Steps counselling service for suicidal ideation and behavior. 2. Frontline Staff—Staff training Evaluation 3. Counsellors—Post-Evaluation Counsellor Focus Group Participants included members of the Sure Steps Counselling Service. These counsellors were previously trained in the CAMS intervention model, in a two-day off-site training, and subsequently implemented this model with several clients within the service.	The Collaborative Assessment and Management of Suicidality (CAMS) is a flexible therapeutic framework in which clients who are experiencing suicidal ideation work collaboratively with the practitioner to assess the client’s suicidal risk and use that information to plan and manage suicide-specific, “driver-oriented” treatment (Jobes et al., 2007). At the core of the CAMS Approach is the Suicide-Status form (SSF), a measure of client current suicide risk and potential for suicide behavior. Staff Questionnaire: The sample included 30 CAMS training participants (24 women and 6 men, across age categories 18–24 to 55–64) at one training event. Semi-structured follow-up phone interviews consisted of 10 randomly chosen CAMS training participants. Staff training involved participants engaging in a 3-h online learning module, a one-day live role play workshop, and follow up case consultation phone calls. Training detailed the Collaborative Assessment and Management of Suicidality model.	The aim of the study was to evaluate the CAMS approach in terms of ease of training, implementing the intervention and most importantly its effectiveness for reducing suicidal thoughts and behavior among the homeless population.	1. Clinical intervention individuals identified within the Dublin Simon Community’s services as having a suicidal risk or who had had previously indicated suicidal ideation and behavior were subsequently referred to the CAMS Intervention Team. The Suicide Status Form (SSF) was used for the clinical assessment and tracking of suicidal clients from the initial to the final intervention session. Of interest in the current analysis were the SSF ratings themselves and their supplementary qualitative responses, Reasons for Living and Reasons for Dying indicated by clients, as well as indications of previous suicidal behavior. Clients attended sessions in various locations attached to the Sure Steps Counselling Service across Dublin city. Initial sessions included the completion of pre-session SSF forms while clients engaged with one of four members of the counselling team. Successful completion of the intervention and the resolution of suicidal risk was indicated by three consecutive sessions of no suicidal thoughts, feelings, and behaviors. 2. The staff training evaluation comprised a set of pre-post questions that were presented to participants via an online questionnaire and an on-site questionnaire. The questionnaire-based evaluation was followed up with brief semi- structured in person interviews two months after the training. Interview questions focused on experiences with using the training content in practice as well as the strengths and limitations of the training itself. 3. Post-Evaluation Counsellor Focus Group Participants were invited to attend the focus group within a suitable location attached to the Sure Steps Service. A topic guide was generated to cover various aspects of the CAMS training and implementation process. The focus group was audio recorded and digitally transcribed for analysis.
Lynn et al., 2014	Ohio, USA The study was conducted with youth residing in family housing shelters in New York City.	Randomized control trial	Participants in the HOPE (HIV Outreach for Parents and Early Adolescents) Programme, who indicated suicidal ideation and behavior were included in the study. The sample consisted of 28 young adolescents (11–14 years of age). The sample was drawn from a larger study of 204 urban parents and their school aged children.	The study contrasts two prevention strategies: (1) HOPE Family Programme an intensive family strengthening intervention to build communication, parental monitoring, and supervision skills and assist parents to manage stressful situations inside and outside of the shelter. (2) HOPE Health Educational Programme provides informational sessions pertaining to methods of prevention of HIV/AIDS and sexually transmitted infections, the effects of the use of illicit substances, and normative adolescent changes For both programmes a social worker was present to provide clinical support to youth and parents as needed. The primary facilitators for both programmes were community members with five years or more experience in HIV prevention services.	The aim of the study was to investigate prevalence of suicidal ideation, the relationship between various risk factors, and the impact of participation in family-based HIV prevention programmes upon self-harm among a sample of adolescents residing in urban homeless shelters with their families	Of these 204 families residing in the shelter, 48.5% (n = 99) were assigned to the HOPE Family Programme, and 51.5% (n = 105) were assigned to HOPE Health within randomly assigned shelters with one programme offered in each shelter. Changes in suicidal ideation from pre-test to post-test was compared across two group approaches to delivering HIV prevention. The following measures were also administered: 1. Youth and family demographics variables; 2. Shelter relater characteristics; 3. Within family supports, 4. The Parenting Skills Questionnaire; 5. Violence Exposure via a 9-item risk subscale from the Family Stress Scale; 6. Caregivers and youth were asked to identify whether they or another member of their family experienced a series of potentially stressful events (yes/no); 7. Substance use was measured via three items from Monitoring the Future survey; 8.Youth suicidal ideation was measured via a single item on the Child Depression Inventory.
Slesnick et al., 2019	Florida, USA Drop-in center for homeless youth in a large Midwestern city,	Randomized control trial	The sample consisted of 150 young adults who were homeless. Participant’s age ranged from 18–24 years. Participants were referred if they had one or more episodes of severe suicidal ideation in the past 90 days.	Cognitive Therapy for Suicide Prevention (CTSP) developed by Wenzel et al. (2009). During the initial phase of treatment (sessions 1–3) clients are educated about the cognitive model and a cognitive case-conceptualization is developed to guide the intervention based on client’s individual risk-factors and experiences. Specifically, automatic thoughts, core beliefs, and key life events associated with suicidal behaviors and thoughts are identified. The middle phase of treatment (sessions 4–7) focuses on both cognitive restructuring and behavior change through a variety of cognitive techniques designed to address suicide-specific risk factors. The objective of the later sessions (8–10) is to prevent relapses through practicing the newly acquired skills through a guided imagery process. TAU sessions offered by therapists at the drop-in center are unsystematic and not manualized. Therapist training consisted of readings and a three-day on-site training in the intervention, including role play exercises by one of the original developers. Ongoing weekly telephone/skype supervision. Therapists were independently licensed master’s level counselors/social workers hired from the drop-in center and all therapy sessions were recorded.	The aims of the study were to: (1) assess the viability of recruiting the intended sample of currently suicidal youth; and (2) assess the feasibility of engaging and retaining non-treatment seeking suicidal youth in the suicide prevention intervention; (3) assess the efficacy of the suicide prevention intervention, as compared to treatment as usual (TAU) provided at a local drop-in center.	Youth were approached at the drop-in center and screened for interest in the study and suicidal ideation by a research assistant. Interested youth with current suicidal ideation reviewed and signed an informed consent statement and were administered the SCID section on psychosis and the SSI–W to determine formal eligibility. Individuals who met the criteria for participation continued with the assessment battery. Upon completion of the baseline assessment, youth were randomly assigned using a computerized randomization programme to either CTSP + Treatment As Usual (TAU) (n = 73) or TAU (n = 69). An intent- to-treat design was used in which all youth, regardless of participation, were tracked for follow-up assessments. Follow-up assessments occurred at 3-months (T1, retention rate 89.4%), 6-months (T2, 86.6%), and 9-months (T3, 85.9%) post-baseline. Data from the additional measures were analyzed; Beck Depression Inventory I; Social Network Interview; The Interpersonal Needs Questionnaire.
Wu et al., 2020	Florida, USA Recruited from the only drop-in center for homeless youth in a large Midwestern city	Randomized control trial	The sample consisted of 150 young adults who were homeless. Participant’s age ranged from 18–24 years. Participants were referred if they had one or more episodes of severe suicidal ideation in the past 90 days.	Cognitive Therapy for Suicide Prevention (CTSP) developed by Wenzel et al. (2009) was employed. During the initial phase of treatment (sessions 1–3) clients are educated about the cognitive model and a cognitive case-conceptualization is developed to guide the intervention based on client’s individual risk-factors and experiences. Specifically, automatic thoughts, core beliefs, and key life events associated with suicidal behaviors and thoughts are identified. The middle phase of treatment (sessions 4–7) focuses on both cognitive restructuring and behavior change through a variety of cognitive techniques designed to address suicide-specific risk factors. The objective of the later sessions (8–10) is to prevent relapses through practicing the newly acquired skills through a guided imagery process. Intervention fidelity was assessed using the Cognitive Therapy Rating Scale. Two therapists received CTSP training and weekly supervision with audiotape reviews. The off site supervisor reviewed recordings.	The aim of the study was to investigate the moderating relations of family network satisfaction on the treatment effects of CTSP, as well as the prospective associations among perceived burdensomeness, belonging, and suicidal ideation	Youth were approached at the drop-in center and screened for interest in the study and suicidal ideation by a research assistant. Interested youth with current suicidal ideation reviewed and signed an informed consent statement and were administered the Structured Clinical Interview for DSM-5 disorders psychotic screening (SCID) section on psychosis and the Scale for Suicide Ideation-Worst Point (SSI–W) to determine formal eligibility. Individuals who met the criteria for participation continued with the assessment battery. Upon completion of the baseline assessment, youth were randomly assigned using a computerized randomization programme to either CTSP + Treatment As Usual (TAU) (n = 73) or TAU (n = 69). An intent- to-treat design was used in which all youth, regardless of participation, were tracked for follow-up assessments. Follow-up assessments occurred at 3-months (T1, retention rate 89.4%), 6-months (T2, 86.6%), and 9-months (T3, 85.9%) post-baseline. Data from the additional measures were analyzed; the Beck Hopelessness Scale; Beck Depression Inventory I; the Inventory of Cognitive Distortions;

**Table 4:** Effectiveness of Interventions.

Author Year of Publication	Effectiveness of Interventions in Reducing Suicidal Ideation/Behaviour	Length of Intervention	Tools Used to Assess Suicide Risk	Focus of the Intervention
Adams et al., 2018	CAMS intervention data shows a clinical reduction in self-reported SSF and suicidal risk ratings at final CAMS sessions in comparison to initial sessions (N = 4).	Three to thirteen sessions were offered to clients in this study.	The Suicide status form (SSF). The SSF collects both qualitative and quantitative data from the client.	The collaborative approach of the intervention used in this study is emphasized as one key mechanism of change in challenging clients’ reasons for living and reasons for dying. The most reported reason for living and dying was relationships/others.
Lynn et al., 2014	Of the 28 youth with suicidal ideation at baseline, 64% (n = 18) indicated changes in suicidal ideation and then subsequently indicated no ideation at post-test. The remaining 36% (n = 10) of youth reported suicidal ideation at baseline, also reported suicidal ideation at post-test.	The HOPE family intervention consisted of eight one hour sessions. The HOPE Health programme consisted of three 2-h sessions.	The Child Depression Inventory (Finch, Saylor, Edwards, and McIntosh, 1987)	The HOPE Family Programme was 13 times more likely to report a decrease of suicidal ideation compared with the education only approach. The HOPE family programme included a great emphasis on family processes, communication and coping skills. Findings also indicated that youth who reported using at least one substance within the prior 30 days were 11 times more likely to report no changes in suicidal ideation relative to baseline.
Slesnick et al., 2019	The follow-up rates were 87%, 87% and 87% at the 3-, 6-, and 9-month follow-up in the CTSP + TAU condition, and 92%, 85%, and 87% in the TAU condition, respectively.	The CTSP consisted of an average of 10 sessions. An option of nine additional maintenance sessions. TAU sessions offered by therapists at the drop-in center were reported to be unsystematic and not manualized. With sessions lasting usually 50 min.	Scale for Suicide Ideation-Worst Point (SSI-W; Beck et al. 1999). The Structured Clinical Interview for DSM-5 disorders psychotic screening (SCID) section on psychosis.	High family network satisfaction enhanced treatment effects of CTSP regarding suicidal ideation and thwarted belongingness. Family network satisfaction moderated the relation between thwarted belongingness and suicidal ideation.
Wu et al., 2020	Significant decline over time in the whole sample in suicidal ideation.	The CTSP consisted of an average of 10 sessions. An option of nine additional maintenance sessions. TAU sessions offered by therapists at the drop-in center were reported to be unsystematic and not manualized. With sessions lasting usually 50 min. Duration 9 months in total	Scale for Suicide Ideation-Worst Point (SSI-W; Beck et al. 1999). The Structured Clinical Interview for DSM-5disorders psychotic screening (SCID) section on psychosis. 9-month period (baseline as well as 3-month, 6-month, and 9-month follow-up assessments).	Among youth with high family network satisfaction, CTSP was associated with lower suicidal ideation at T3 at a trend level, the effect was not significant for youth with low family network satisfaction.

## Data Availability

Not applicable.

## Supplementary Materials

The following are available online at https://www.mdpi.com/article/10.3390/ijerph18136729/s1. Supplementary Materials File S1: Scoping review protocol. Supplementary Materials File S2. Mixed Methods Assessment Tool.
