# Supplementary material for: Scoping Review: Suicide Specific Intervention Programmes for People Experiencing Homelessness
Source: Int J Environ Res Public Health. 2021 Jun 22;18(13):6729. doi: 10.3390/ijerph18136729 (PMC8297158; doi:10.3390/ijerph18136729)
Supplement: Supplementary file 1 [file ijerph-18-06729-s001.zip › ijerph-1193920-supplementary.pdf]

Supplementary Material

[Supplementary material file S1: Scoping review protocol}

**Proposed review questions**	
What is the extent, scope, effectiveness and context of suicide prevention intervention programmes for individuals who are homeless.	
Rationale for this scoping review/ working abstract	
Introduction<br>Suicide is a complex phenomenon emerging from a dynamic interaction of biological, psychological, social and cultural factors (Quarshie, Osafo, Akotia & Peprah, 2015). The homeless population are among the most vulnerable groups to experience suicide ideation and behaviour, with studies showing a remarkably higher magnitude of suicidal ideation and attempt among the homeless population compared to the general population (Ayano et al., 2019). This highlights the need for evidence-based suicide prevention interventions and related assessment tools for mental health professionals working in this sector. However, there is limited research into which components of interventions, in particular those delivered in clinical settings, are most effective in reducing suicidal ideation and behaviour among the homeless population. The aim of this scoping review is to characterize the state-of-the science in the area of suicide prevention for homeless individuals, uncover best practices of prevention, counselling and treatment and identify any further research which may need to be conducted.<br><br>Methods<br>This scoping review will follow Arksey and O'Malley's [32] five-stage scoping review framework will be followed: (1) identifying the research question; (2) identifying the relevant literature; (3) selecting the studies; (4) charting the data and (5) collating, summarising and reporting the results and be guided by the PRISMA guidelines. An additional step, following the charting of the data, was to consult and expert panel, to validate the findings from the search. The authors consulted with a librarian to determine a search strategy, ensure all possible search terms were included and to identify all possible sources of articles including the location of grey literature and unpublished theses. Four database searches; Psychinfo, Pubmed, Cinhal and Embase were searched.	
Aim of this systematic review	
The aim of a scoping review is to map the existing literature on suicide prevention for individuals who are homeless to determine the extent, scope effective and context in which these approaches have been implemented.	
Specific Objectives	
1. To identify the research conducted in this area, and to identify gaps, if any, among the literature and document whether further research may need to be conducted.<br>2. To assess the quality of studies which have been conducted.<br>3. To identify best practices of reducing suicidal ideation and behaviour among the homeless population.<br>4. To identify which components of interventions are most effective in reducing suicidal ideation and behaviour among the homeless population.	
Population:	The homeless population
Intervention:	Suicide specific clinical intervention<br>Psychological Treatments for Suicide<br>Crisis intervention<br><br>An intervention or programme which has been designed specifically to reduce suicidal ideation and behaviour.
Outcome:	The outcome of interest is suicidal ideation behaviour following treatment. Outcome measures are envisioned to be heterogenous across studies.
**Search strategy**	

In line with the preferred reporting items for systematic review and meta-analysis protocols (PRISMA-P), the search strategy and search terms are outlined below (Moher et al., 2015).

Databases

Grey literature – Open Grey and Bielefeld Academic Search Engine were used to search for grey literature.

The PubMed search terms will also be mapped to Medical Subject Headings (MeSH) terms, and similar terms in EMBASE and PsycINFO and CINHL. References of articles identified will be used to source additional studies.

Search terms

The authors consulted with a librarian to determine a search strategy, ensure all possible search terms were included and to identify all possible sources of articles including the location of grey literature and unpublished theses. The PubMed, PsychInfo, CINHAL databases were searched for records up to April 2020. Three concepts were identified "intervention", "homelessness" and "suicide". Search terms were as follows: ("suicide"* OR "self-harm" OR "deliberate self-harm" OR "self-injurious behaviour*" OR "suicidal ideation" OR "mentally ill" OR attempted suicide") AND ("homeless*" OR "no fixed abode" OR "rough sleeper" OR "homeless person" OR "person, homeless" OR "street people" OR "people, street") AND ("suicide intervention*" OR "psychotherapy" OR "cognitive psychotherapy" OR "treatment" OR "crises intervention service*" OR "evidence based practice" OR "mental health service" OR "management" OR "measurement" OR "assessment" OR "cognitive therapy" OR "health care utilization" OR "emergency services" OR "collaborative care" OR "prevention" OR "suicide prevention") (note: * indicates a wildcard).

Data extraction (selection and coding)

Title and abstracts will be assessed for eligibility by one reviewer. Full papers will then be assessed independently by two reviewers, any discrepancies will be resolved through discussion, with reference to a third reviewer if necessary.

The articles will be identified were charted according to the following headings:

- Author(s),
- year of publication,
- study location
- Study design
- Intervention description
- Study sample or population characteristics
- Aims of the study
- Methodology
- Outcome data on suicide deaths, suicide attempt, suicidal ideation, suicide-related behaviour, and/or self-harm at the point of post-intervention and longest follow-up.

Important results

Mixed Methods Assessment Tool Quality assessment

Each study which has met inclusion criteria will be assessed by two reviewers independently using the MMAT for quality assurance.

Methods

The Preferred Reporting Items for Systematic Reviews and Meta-Analyses (PRISMA-SCR) guidelines (Tricco et al 2018) will be followed. Based on the reference list of relevant articles from the database search, additional articles will be screened for inclusion. Grey literature (case reports, conference abstracts, theses, reviews, and editorials) will be considered and subjected to the predefined inclusion.

The five steps outlined by Arksey & O'Malley's [32] were used to carry out the scoping review.

1. Outline the research question
2. Identifying relevant studies
3. Study selection
4. Charting the data
5. Collating, summarising and reporting the results.
6. Validate findings with content experts.
7. Discussion

[Supplementary Material. File S2. Mixed Methods Assessment Tool]

Study ID	*Screening questions*		*Quantitative*									
			**Randomised controlled trial**					**Mixed methods**				
	S1	S2	2.1	2.2	2.3	2.4	2.5	5.1	5.2	5.3	5.4	5.5
Adams et al. (2018)	Y	Y						Y	Y	Y	N	Y
Lynn et al. (2014)	Y	Y	CT	Y	Y	CT	Y					
Slesnick et al. (2020)	Y	Y	Y	Y	Y	N	Y					
Wong et al. (2019)	Y	Y	Y	Y	Y	N	Y					

Y=yes, N=no, CT= can't tell

Screening Questions

- S1 Are there clear research questions?
- S2 Do the collected data allow to address the research questions?

Randomised Controlled Trial

- 2.1 Is randomization appropriately performed?
- 2.2 Are the groups comparable at baseline?
- 2.3 Are there complete outcome data?
- 2.4 Are outcome assessors blinded to the intervention provided?
- 2.5 Did the participants adhere to the assigned intervention?

Mixed methods

- 5.1 Is there an adequate rationale for using a mixed methods design to address the research question?
- 5.2 Are the different components of the study effectively integrated to answer the research question?
- 5.3 Are the outputs of the integration of qualitative and quantitative components adequately interpreted?
- 5.4 Are divergences and inconsistencies between quantitative and qualitative results adequately addressed?
- 5.5 Do the different components of the study adhere to the quality criteria of each tradition of the methods involved?
